# Neurotoxicity including posterior reversible encephalopathy syndrome after initiation of calcineurin inhibitors in transplanted methylmalonic acidemia patients: Two case reports and review of the literature

**DOI:** 10.1002/jmd2.12088

**Published:** 2020-01-22

**Authors:** Femke Molema, Monique Williams, Janneke Langendonk, Sarwa Darwish‐Murad, Jacqueline van de Wetering, Ed Jacobs, Willem Onkenhout, Esther Brusse, Anke van der Eerden, Margreet Wagenmakers

**Affiliations:** ^1^ Department of Pediatrics, Center for Lysosomal and Metabolic Disease Erasmus ‐ Sophia Children's Hospital, University Medical Center Rotterdam The Netherlands; ^2^ Department of Internal Medicine, Erasmus University Medical Center Center for Lysosomal and Metabolic Disease Rotterdam The Netherlands; ^3^ Department of Gastroenterology and Hepatology Erasmus University Medical Center Rotterdam The Netherlands; ^4^ Department of Internal Medicine Erasmus University Medical Center, Nephrology and Transplantation, Rotterdam Transplant Group Rotterdam The Netherlands; ^5^ Department of Clinical Genetics Erasmus University Medical Center Rotterdam The Netherlands; ^6^ Department of Neurology Erasmus University Medical Center Rotterdam The Netherlands; ^7^ Department of Radiology Erasmus University Medical Center Rotterdam The Netherlands

**Keywords:** calcineurin inhibitors, liver and/or kidney transplantation, methylmalonic acidemia, neurotoxicity, posterior reversible encephalopathy syndrome/PRES

## Abstract

**Introduction:**

New neurological symptoms in methylmalonic acidemia (MMA) patients after liver and/or kidney transplantation (LKT) are often described as metabolic stroke‐like‐events. Since calcineurin inhibitors (CNIs) are a well‐known cause of new neurological symptoms in non‐MMA transplanted patients, we investigated the incidence of CNI‐induced neurotoxicity including posterior reversible encephalopathy syndrome (PRES) in post‐transplanted MMA patients.

**Methods:**

We report the two MMA patients treated with LKT in our center. Additionally, we performed a systematic review of case reports/series of post‐transplanted MMA patients and determined if CNI‐induced neurotoxicity/PRES was a likely cause of new neurological symptoms. Definite CNI‐induced neurotoxicity was defined as new neurological symptoms during CNI treatment with symptom improvement after CNI dose reduction/discontinuation. PRES was defined as CNI‐induced neurotoxicity with signs of vasogenic edema on brain magnetic resonance imaging (MRI)‐scan post‐transplantation.

**Results:**

Our two MMA patients both developed CNI‐induced neurotoxicity, one had PRES. In literature, 230 transplanted MMA patients were identified. Neurological follow‐up was reported in 54 of them, of which 24 were excluded from analysis since no anti‐rejection medication was reported. Thirty patients, all using CNI, were included. Sixteen patients (53%) had no new neurological symptoms post‐transplantation and five patients (17%) had definite CNI neurotoxicity of whom two had PRES. Including our cases this results in a pooled incidence of 22% (7/32) definite CNI neurotoxicity and 9% PRES (3/32) in post‐transplanted MMA patients on CNI.

**Conclusion:**

In MMA post‐transplanted patients with new neurological symptoms CNI‐induced neurotoxicity/PRES should be considered. Early recognition of CNI‐induced neurotoxicity is essential to initiate dose reduction/discontinuation of CNI to minimize persistent neurologic damage and improve outcome.

**Concise one sentence take home message:**

In all post‐transplanted MMA patients with new neurological symptoms CNI‐induced neurotoxicity/PRES should be considered, and directly reducing the dose/discontinuation of CNI is essential.

**Abbreviations**
CNI: calcineurin inhibitor
CSF: cerebrospinal fluid
DWI: Diffusion‐weighted imaging
LKT: liver and/or kidney transplantation
mma: methylmalonic acid
MMA: methylmalonic acidemia
MMF: mycophenolate mofetil
MRI: magnetic resonance imaging
POD: post‐operative day
PRES: posterior reversible encephalopathy syndrome

## 1 INTRODUCTION

Methylmalonic acidemia (MMA) is a severe rare inborn error of metabolism, belonging to the organic acidemias. MMA leads to increased levels of methylmalonic acid (mma). Isolated MMA is caused by complete (*mut* ^0^) or partial (*mut* ^−^) deficiency of the mitochondrial enzyme methylmalonyl‐CoA mutase (MUT) (OMIM #251000) or by deficient synthesis of the MUT‐cofactor adenosylcobalamin (CblA (OMIM #251100) or CblB [OMIM #251110]).^1^ While survival of MMA patients has greatly improved over the past decades with conventional treatment strategies,^2^, ^3^ patients continue to develop serious long‐term complications,^4^ including renal insufficiency and neurological complications, such as developmental delay, seizures, and metabolic stroke.^5^ Furthermore, patients have an impaired quality of life.^6^

Since the prognosis of MMA patients is often poor, liver and/or kidney transplantation is performed with increased frequency.^7^, ^8^ Although the liver is the main site of MUT enzyme expression, the enzyme is expressed in other tissues as well,^9^ including the kidneys and in lesser extent the muscles and brain.^10^ Hence, liver and/or kidney transplantation does not fully restore MUT enzyme activity. The outcome of transplantations in MMA patients varies and there are multiple reports of patients who developed new neurological complications after transplantation.^11^, ^12^ Concerns about new neurological complications after transplantation is well described and it is mentioned in a recent guideline on organic acidurias.^5^ The new neurological complications after transplantation can be due to deficient MUT activity in non‐transplanted tissues leading to high mma levels in cerebrospinal fluid^13^, ^14^ and cerebral tissues or to metabolic encephalopathy during decompensations.

However, new neurological complications are also a disturbing and relatively common phenomena in non‐MMA patients after organ transplantation (occurring in 15%‐40% of patients).^15^, ^16^, ^17^ These neurological complications can be caused by a variety of factors including immunosuppressive medication (especially calcineurin inhibitors [CNI], such as tacrolimus and cyclosporine).^17^ CNI‐induced neurotoxicity in non‐MMA organ transplanted patients can present with a variety of symptoms such as confusion, tremor, seizures, cerebral hemorrhage, ischemic stroke, and posterior reversible encephalopathy syndrome (PRES).^15^, ^18^, ^19^, ^20^ In CNI‐induced PRES, the brain magnetic resonance imaging (MRI)‐scan typically shows bilateral areas of vasogenic edema, which may be located in the cortex and/or subcortical white matter of brain areas; supplied by posterior circulation. However, an atypical distribution pattern is not infrequently observed, for example, involving the basal ganglia and/or the brain stem,^20^ with or without atypical imaging findings (contrast enhancement, hemorrhage or restricted diffusion).^18^

Recently, two adult MMA patients received combined liver/kidney transplantation in our center. They were the first transplanted MMA patients in our center, and both patients had new neurological symptoms within months post‐transplantation, which we attributed to CNI‐induced neurotoxicity. The aim of this study was to investigate the occurrence of CNI‐induced neurotoxicity in liver and/or kidney transplanted MMA patients. Therefore, we describe the two transplanted MMA patients in our center and performed a systematic review of previously published case reports and case series of MMA patients following liver and/or kidney transplantation. We retrospectively reviewed if neurological symptoms could be attributed to CNI‐induced neurotoxicity. Furthermore, we calculated the pooled incidence of CNI‐induced neurotoxicity, including PRES, in MMA patients in our center and from literature.

## 2 METHODS

To identify all published case reports and case series of transplanted MMA patients, we performed a systematic search of MEDLINE and EMBASE databases on the 11th of September 2018. Search terms were “mma OR (methylmalonic AND acidemia) OR (methylmalonic AND aciduria) OR (methylmalonic AND acidaemia) OR mcm OR (methylmalonyl AND coa AND mutase) OR mut OR (mcm AND deficiency) OR (acidemia AND methylmalonic) OR (cblb AND type) OR (cblb AND disease) OR (cblb AND disorder) OR (cbla AND type) OR (cbla AND disease) OR (cbla AND disorders) OR mut‐ OR mut0 OR mmaa OR mmab AND transplantation OR transplant OR grafting OR graft OR implantation OR implant AND liver OR hepatic OR kidney OR renal OR combined OR (liver AND kidney) OR clkt” in Embase and Pubmed. Exclusion criteria were another disease than MMA and in vitro or animal studies. Duplicate patients identified by the same author(s) and same age at transplantation (when reported) and/or mutation (when reported) were excluded. Informed consent of the two Erasmus MC patients was obtained.

### 2.1 Definitions

We defined the condition of “definite CNI‐induced neurotoxicity” as new neurological symptoms that started while the patient was using CNI, as anti‐rejection medication, within the first year after transplantation and improvement following dose reduction or discontinuation. We attributed new neurological symptoms after transplantation to “probable CNI induced neurotoxicity” as (a) patients with new seizures, unlikely due to another cause than CNI, within 1 year after transplantation (in case time period was mentioned) without reported anti‐rejection medication or (b) newly acquired symptoms, unlikely due to another cause than CNI, within 1 year after transplantation while on CNI with unclear outcome reported of neurological symptoms. We attributed “CNI‐induced PRES” to “definite CNI‐induced neurotoxicity” in combination with (1) brain MRI‐scan abnormalities, with typical imaging findings (signs of vasogenic edema) with a typical (in parieto‐occipital and/or posterior frontal cortex and/or subcortical white matter) or an atypical distribution (in other brain areas such as brain stem, basal ganglia, subcortical, or cortical frontal regions without a posterior predominance), with or without atypical imaging findings (such as contrast enhancement, hemorrhage and or restricted diffusion on brain MRI‐scan) and either 2a) improvement of brain MRI‐scan findings on follow‐up or, in case of unavailability of follow‐up MRI 2b) return to baseline neurological status after dose reduction or discontinuation of CNI.^16^, ^18^, ^21^

### 2.2 Data analysis from the literature

Characteristics of all described outcome parameters of each reported case were derived from the reports that were found via the literature search. Three reviewers (F.M., M.W., and M.W.) independently judged whether or not the cases were described in enough detail to conclude that they had (a) generally reported follow‐up after solid organ transplantation, (b) reported neurological follow‐up. Subsequently, the same reviewers independently decided in which group the patients with new neurological symptoms should be included, that is, (a) patients without reported post‐transplantation anti‐rejection medication, (b) patients with reported anti‐rejection medication, either with or without neurological symptoms. Finally, the reviewers retrospectivity attributed new neurological symptoms to (a) “definite CNI‐induced neurotoxicity,” subdividing them into patients with and without PRES, (b) “probable CNI‐induced neurotoxicity,” (c) neurological symptoms due to another cause (such as stroke‐like event). Thereafter consensus was made on those cases that were non‐conclusive.

### 2.3 Analysis of FGF‐21 concentrations in the two cases from the Erasmus MC

We measured serum levels of FGF‐21 levels in the two patients transplanted in our center on several time points before and after transplantation and during neurological events. All were measured using a commercial enzyme‐linked immunosorbent assay (ELISA) kit (Millipore) according to local protocol, as reported in Molema et al.^22^

### 2.4 Analysis of MRI findings in the two cases from our center

The MRI‐scans, existing of T1‐weighted, T2‐weighted and diffusion‐weighted imaging (DWI) before, and T1‐weighted and 3D FLAIR imaging after gadolinium injection, were performed in a clinical setting. The initial scan of case 1 additionally included susceptibility weighted imaging and contrast enhanced MR angiography. The neuroradiologist (AE) from the Erasmus MC performed a second reading of the MRI‐scans, taking into account the original radiological reports.

### 2.5 Statistical analysis

Descriptive parameters (frequencies, median and range) were used as outcome measurements in this study. The pooled incidence of CNI‐induced neurotoxicity, including PRES, was calculated by combining the patients from literature and the patients from our center.

## 3 RESULTS

### 3.1 Case reports from our center

#### 3.1.1 Case 1

A 29‐year‐old female patient was diagnosed with MMA at the age of 4.5 months while presenting with coma, vomiting and hepatomegaly. The patient was non‐vitamin b12 responsive and the MMA was caused by a homozygous frameshift mutation in the MUT gene (c.1311_1312insA) (Table 1). At the age of 28 years, she had been admitted to the hospital 58 times for metabolic derangements. She had a mild intellectual disability; she worked as a shop‐assistant. She had developed visual loss at 12 years of age and progressive renal insufficiency. At age 28 years she required hemodialysis and she opted for a combined LKT. There were no peri‐operative complications. She was placed on an immunosuppressive regime of tacrolimus, prednisone and mycophenolate mofetil (MMF) and her diet was liberated to a non‐restricted diet. Of the previous medication, only carnitine and vitamin B12 supplementation were continued. She recovered well without any metabolic disturbance.

**Table 1:** Patient characteristics of patients with CNI‐induced neurotoxicity

Case	Case 1	Case 2	Giusanni	Niemi	Vernon	Mc Guire	Nagarajan
Year publication, gender	2019, f	2019, f	2016, m	2015, m	2014, f	2008, m	2005, m
Age onset, age at diagnosis	5 mo, 5 mo	12 days, un	un, <1 y	both un	3 mo, 9 mo	<3 mo, 3 mo	1 week, 1 week
Genotype	Mut0 c.1311_1312insA, p.Val438Serfs*3 non‐vitamin B12 responsive	Mut0 c.2078delG, p.G693Dfs*12 non‐vitamin B12 responsive	un	un	Mut0 c.2053dupCTC p.685insL non‐vitamin B12 responsive	Mut0	Mut0
Neurologic complications pre‐Tx	None	None	un	un	Mild choreoathesosis due to bilateral globus pallidus infarction	Aphasic and difficulty ambulating due to weakness and tremors	Deterioration with dystonia, muscular weakness and wheelchair bound
Other complications pre‐Tx	Impaired vision due to optic nerve infarction, age 12 y ESRD^a^, age 18y, she required hemodialysis	Near blind from sudden onset optical atrophy, age 17 y Chronic renal insufficiency age 10y, stage IV age 18y	un	un	Acute bilateral opticus neuropathy, Chronic kidney disease^b^	Renal insufficiency, frequent metabolic decompensations	Pancreatitis
Development pre‐Tx	Normal: WAIS‐IV performed at age 27y: normal with mild expressive language problems	Normal	un	un	Normal	un	Cognitive development delay, decreased motor skills
Age Tx	28 y	19 y	6 y	un	28 y	5 y	21 y
Duration follow‐up	14mo	8 mo	un	un	un	10 mo	1 y 6 mo
Tx	Combined LKT	Combined LKT (lost renal Tx)	Combined LKT	Liver	Combined LKT	Combined LKT	Combined LKT
Medication after Tx	Tacrolimus, MMF, prednisone	Tacrolimus, MMF, prednisone	Cyclosporine, prednisone	Tacrolimus, prednisone, azathioprine	MMF, prednisone, basiliximab, POD 6 tacrolimus	Tacrolimus and steroids	Tacrolimus, sirolimus, prednisone
Start neurological symptoms post‐Tx	3 mo	22 days	10 days	12 days	28 days and 48 days	Weeks	un
Neurological symptoms	Bradyphrenia, severe ataxia, behavioural changes	Seizures	Seizures	Seizures	Seizures	Altered mental status, aphasia, hallucinations, seizures, tremor	Altered mental status, tremors
Tacrolimus level during neurological symptoms (ng/mL)	10.7	3.1‐5.5^c^	un	High	6.7	<5	5‐7
Mma levels during neurological symptoms (μmol/l)	272 in plasma, 728 in the cerebrospinal fluid	268 in plasma	un	<343 in plasma	un	<500 in plasma	<500 in plasma
Final consensus diagnosis	CNI‐induced PRES	Definite CNI‐induced neurotoxicity	CNI‐induced PRES	Definite CNI‐induced neurotoxicity	CNI‐induced PRES	Definite CNI‐induced neurotoxicity	Definite CNI‐induced neurotoxicity

Abbreviations: CNI, calcineurin inhibitor; combined LKT, combined liver and kidney transplantation; ESRD, end‐stage renal disease; f, female; m, male; mo, months; Tx, transplantation; un, unavailable; y, year.

a Despite fluid up to 7 L/d via PEG.

b Secondary hyperparathyroidism, hypothyroidism.

c Deliberately lower in setting of combination therapy with MMF due to renal failure.

**Figure 1:**
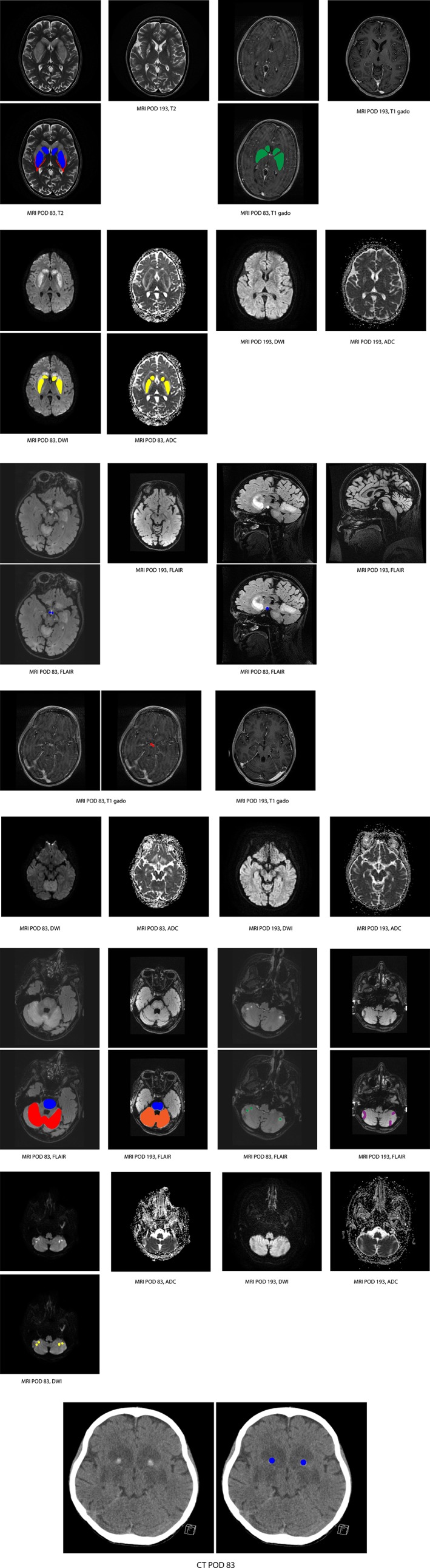
Brain MRI images of case 1 from our center. Post‐operative day (POD) 83 = initial magnetic resonance imaging (MRI) or CT‐scan and POD 193 = follow‐up MRI. FLAIR, fluid attenuated inversion recovery images, acquired after intravenous administration of contrast medium. T1 gado = T1‐weighted image after gadolinium administration; T2 = T2‐weighted images. A, Initial MRI: swelling and T2 hyperintensity of basal ganglia (blue); lentiform fork (red); faint contrast enhancement (green); high intensity on DWI and low/normal ADC in basal ganglia (yellow). Follow‐up MRI: decreased signal abnormalities of basal ganglia, with tissue loss. B, Initial MRI: swelling and T2 hyperintensity of mammillary bodies (blue); avid contrast enhancement (red) and no diffusion restriction. Follow‐up MRI: decreased signal abnormalities, tissue loss. C, Initial MRI: Pons: central T2 hyperintensity (blue). Upper cerebellum, SCA territory: diffuse T2 hyperintensity and swelling (red). PICA and AICA territory: asymmetrical focal T2 hyperintensities (green). Pons and SCA territory: no diffusion restriction (not shown). Focal lesions in PICA and AICA territory: diffusion restriction (yellow). Follow‐up MRI: Pons: central T2 hyperintensity (blue); SCA territory decreased signal abnormalities (orange), diffuse atrophy; PICA and AICA territory: focal tissue loss (purple). D, Calcifications in anterior limb of internal capsule (blue)

Approximately, 2.5 months after the transplantation, she started feeling unwell without objective abnormalities during physical and laboratory examination. After 2 weeks (postoperative day [POD] 83), she presented with headache, ataxia and bradyphrenia. An MMA related metabolic stroke was initially considered, although no metabolic decompensation was present (according to laboratory characteristics), and a strict emergency regime was prescribed. Her brain MRI‐scan (POD83) showed abnormalities: (a) symmetrical T2 hyperintense lesions in basal ganglia (Figure 1A), mammillary bodies (Figure 1B), pons and cerebellum (Figure 1C), (b) swelling and faint contrast enhancement in basal ganglia (Figure 1A), swelling and avid contrast enhancement in the mammillary bodies (Figure 1B) and (c) high signal on DWI with intermediate ADC of the basal ganglia (Figure 1A), no diffusion restriction in the mammillary bodies (Figure 1B), and diffusion restriction of some of the cerebellar lesions (Figure 1C) (Table S1). Due to movement artifacts, no reliable assessment of contrast enhancement of the cerebellar abnormalities was possible (Figure 1C). These findings can be caused by a metabolic (MMA induced) stroke‐like episode but also by PRES. The involvement of the mammillary bodies is atypical for both etiologies. Furthermore, a lentiform fork sign was observed, suggestive of metabolic acidosis, which was biochemically not confirmed. Also, calcifications in anterior limb of internal capsule (Figure 1D) were observed. At POD85 mma was still very low and tacrolimus levels were within the normal non‐toxic range (Table 1). The patient did not improve with the emergency regime and a tacrolimus‐induced PRES was suggested. The tacrolimus was discontinued at POD86 and replaced by everolimus. Within a week the clinical picture of the patient clearly improved, apart from a severe depression (POD 93), which was successfully treated with medication and psychotherapy.

Three months later a new brain MRI‐scan (POD193) showed decreased signal abnormalities, however with tissue loss in basal ganglia, mammilary bodies and diffuse atrophy in SCA territory and focal loss of tissue at the locations of the lesions in the ‐PICA and AICA territories of the cerebellum. At 14 months follow up the ataxia has greatly improved but mild bradyphrenia, orophacial dyskinesia (which is thought to be caused by citolapram) and a mild left‐sided hemiparesis is still present. In conclusion, because the time of onset of the new neurological symptoms after transplantation, the abnormalities on MRI scan and the evident improvement of symptoms after discontinuation of tacrolimus this patient was diagnosed with a CNI‐induced PRES.

##### Laboratory characteristics: mma and FGF‐21

**Figure 2:**
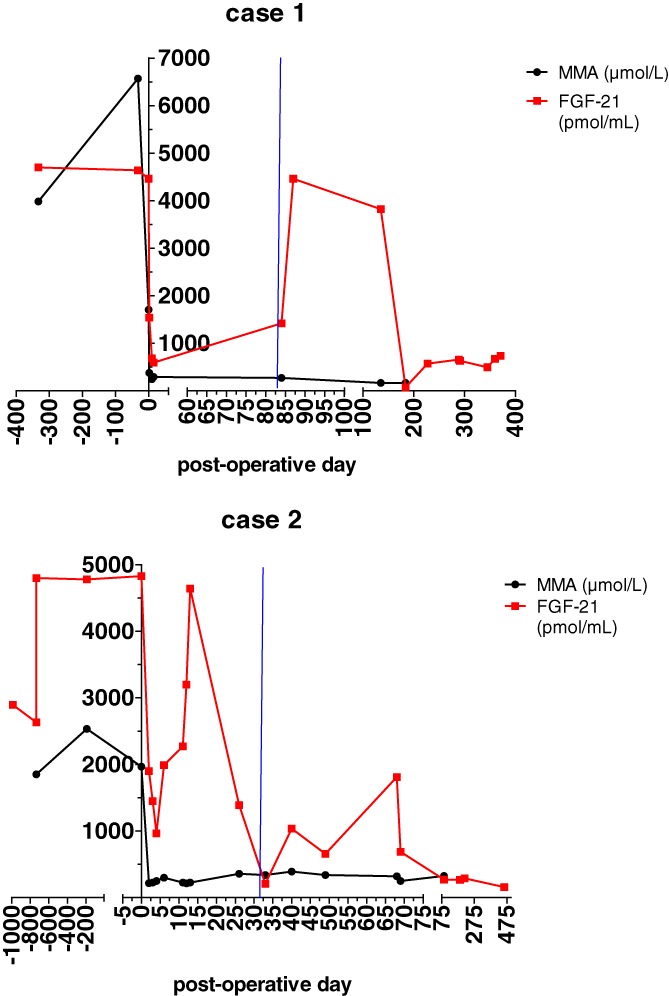
FGF‐21 and mma plasma level before and after transplantation in case 1 (A) and case 2 (B). Blue vertical lines indicate timing of first neurological symptoms due to the CNI

Plasma mma values before transplantation ranged between 3000 and 11 700 μmol/L and after transplantation rapidly declined to 200 and 300 μmol/L on day 4 after transplantation (Figure 2). Although FGF‐21 values, measured retrospectively before transplantation, were very high (>4000 PmoL/mL), they rapidly decreased after transplantation (<700 PmoL/mL). At POD85 when presenting with new neurological symptoms mma was still very low, but there was a significant rise in FGF‐21 plasma levels (retrospectively) (Figure 2). Cerebrospinal fluid (CSF) mma levels (728 μmol/L), measured once during the work‐up of the neurological symptoms, were higher than in plasma with a CSF/plasma mma ratio of 2.7, conform reported in literature.^13^, ^14^ FGF‐21 normalized when she clinically recovered.

3.2

#### 3.1.2 Case 2

A 19‐year‐old female was diagnosed with early‐onset MMA (at the age of 12 days) presenting with vomiting, weight loss and an ammonia level of 172 μmol/L (previously reported in Molema et al as case 12^22^. The MMA was caused by a homozygous frameshift mutation in the MUT gene (c.2078delG), and was non‐vitb12 response (Table 1). At the age of 19 years the patient had been hospitalized a total of 22 times because of metabolic decompensations. At 16 years of age she developed rapid visual loss due to optical atrophy and a progressive renal insufficiency, stage 4 and had to leave school (higher secondary education). Because her clinical condition rapidly deteriorated she received a combined LKT at the age of 19 years and 2 months. Conform case 1; she was placed on an immunosuppressive regime of tacrolimus, prednisone and MMF. While the transplantation itself was uncomplicated the direct postoperative period was complicated by primary kidney graft dysfunction caused by an arterial thrombosis in the anastomosis, which despite a thrombectomy resulted in graft kidney loss, which was removed at POD 2. At POD 7, the patient developed a life‐threatening hemorrhagic shock caused by acute gastrointestinal bleeding (Hb of 2.5 mmol/L) a complication of reflux esophagitis, LA grade C. She recovered well with proton pump inhibitors without neurological complications and the patient received 1 g/kg natural protein per day. On POD 31, she presented with a tonic‐clonic seizure, without signs of metabolic decompensation (according to laboratory characteristics). Triggered by the experience with case 1, CNI‐induced neurotoxicity was immediately suspected even though tacrolimus levels were within normal non‐toxic range and tacrolimus was switched to everolimus on POD31. The brain MRI‐scan performed at POD 32 (Figure S2) showed no signs of PRES nor other signal abnormalities. Coincidentally, a hypoplasia of the vermis cerebelli was found with enlarged fourth ventricle and mildly enlarged supratentorial ventricles. Because of recurrent seizures in the first week after her initial seizure, treatment with levetiracetam was started with good results and no persisting neurological symptoms. However, the patient developed a status epilepticus on POD49, shortly after the everolimus dose was increased, thought to be caused by supra‐therapeutically levetiracetam levels (42 mg/L) (in combination with use of everolimus). She was treated with diphantoine and midazolam, levetiracetam dose was reduced and since everolimus could also be causative everolimus was temporary discontinued. One more episode of three seizures on occurred on POD 68, everolimus was again briefly discontinued. Currently, the patient has a follow‐up of 8 months and she is functioning better than before transplantation (more energy and less nausea) with no lasting new neurological symptoms. She is now treated with everolimus, prednisolone, MMF, levetiracetam (which we plan to discontinue soon), levocarnitine and idebenone. In conclusion, because the time of onset of the new severe epilepsy after transplantation, which resolved after discontinuation of tacrolimus this patient was diagnosed with a CNI‐induced neurotoxicity.

##### Laboratory characteristics: mma and FGF‐21

A significant drop in plasma mma and FGF‐21 after transplantation was seen in both parameters (Figure 2). However, within a week after transplantation the patient had a life‐threatening hemorrhagic shock, in which FGF‐21 increased severely. At her second and third admission with epilepsy, a relatively mild rise in FGF‐21 was seen.

### 3.3 Literature review

**Figure 3:**
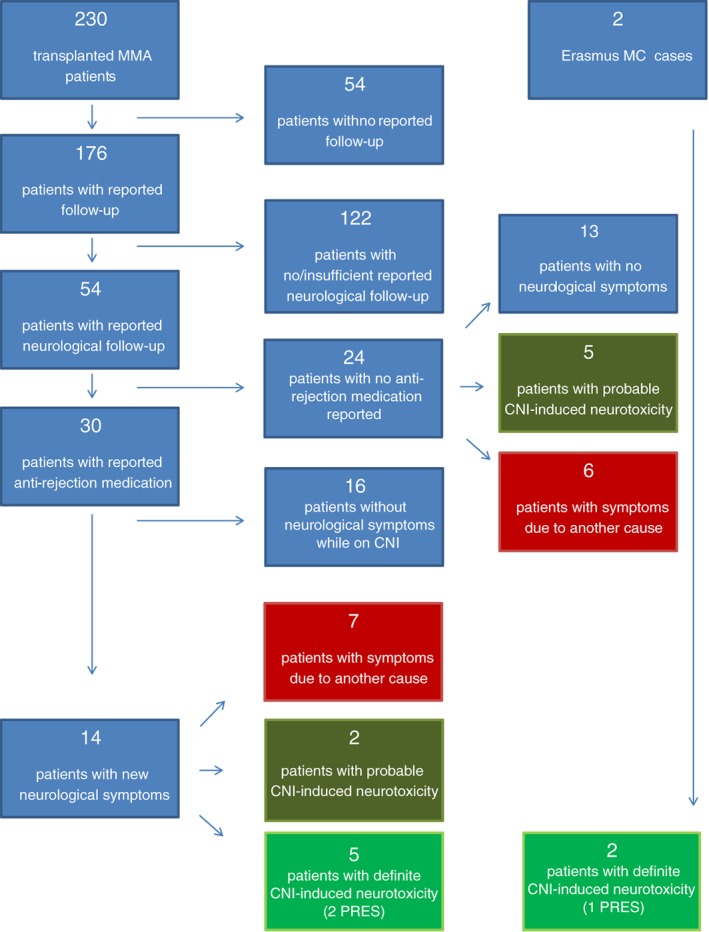
Flow‐chart of literature review and overview of excluded patients (which are further described Table S12A,B and 3A,B). 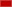 Due to another cause; 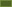 probable CNI‐induced; 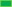 definite CNI‐induced neurotoxicity

A total of 399 unique articles were retrieved and screened on title and abstract. One hundred and twenty‐four of these articles reported a total of 230 transplanted MMA patients. Only 23% of the cases (54/230) explicitly reported neurological follow‐up. Of these 54 patients, 24 were excluded from further analysis because of lack of information on the use of post‐transplant medication (Figure 3). However of these 24 cases, who likely all used a CNI, 5 (21%) had probable CNI‐induced neurotoxicity (Table S2a, dark green boxes Figure 3) and 6 (25%) had acquired new neurological symptoms due to another cause (Table S3a); the remaining patients did not develop new neurological symptoms after transplantation.

Of the 30 cases that were included in our analysis, all were reported to receive a CNI. Sixteen patients (55%) did not develop new neurological symptoms while on CNI, five patients (17%) had definite CNI neurotoxicity (Table 1, bright green boxed Figure 3, (1 patient related to cyclosporine [23], and all others to tacrolimus), two patients (7%) had probable CNI‐induced neurotoxicity and seven patients (23%) developed new neurological symptoms due to another cause (Table [Link], [Link]). Of the two patients with probable CNI‐induced neurotoxicity (dark green boxes Figure 3), one patients did not improve when tacrolimus was switched to cyclosporine (another CNI) and in the other patient no information regarding continuation of CNI was available.

Of the five patients with definite CNI neurotoxicity described in literature three had brain MRI‐scan findings reported.^11^, ^23^, ^24^ Signs of vasogenic edema were found in two patients (Table S1); one of these patients had posterior cerebral involvement. Both patients were defined as having PRES considering the improvement of MRI findings and return of symptoms to baseline neurological status after discontinuing CNI, respectively.^23^, ^24^

### 3.4 Pooled incidence of CNI‐induced neurotoxicity, including PRES

In total, five of 30 patients from the literature, and our two cases have been described in enough detail to conclude that they had CNI‐induced neuro‐toxicity (Figure 3, Table 1) resulting in pooled incidence of 22% (7/32) in MMA patients after solid organ transplantation, and an incidence of neurological symptoms due to other etiology than CNI of 22% (7/32) (Table S3).

Overall, in patients with CNI‐induced neurotoxicity median age at transplantation was 20 years (range: 5‐28 years) and the median follow‐up after transplantation was 1.5 years (range 0.33‐11.5 year). Symptoms occurred between POD10 to 3 months after transplantation (Table 1). Symptoms varied widely (Table 1), with seizures being the most frequently presenting symptom (5/7). The majority of patients had tacrolimus levels within reported therapeutic reference ranges (5/6). Overall, 9% (3/32) of the patients likely had a CNI‐induced PRES identified by brain MRI‐scan.

## 4 DISCUSSION

This is the first systematic review that investigates the frequency of new neurological symptoms in MMA patients after liver and/or kidney transplantation and investigates whether these symptoms could be related to CNI‐induced neurotoxicity, including PRES. Since neurological symptoms are likely to resolve when CNI is timely reduced/discontinued it is essential that physicians treating transplanted MMA patients are aware of CNI‐induced neurotoxicity.

### 4.1 CNI‐induced neurotoxicity, including PRES, in transplanted MMA patients

#### 4.1.1 Clinical findings

According to our findings, the risk of developing CNI‐induced neurotoxicity in transplanted MMA patients seems similar to the risk of neurological symptoms due to other etiologies (22%), such a metabolic stroke‐like episode. Differentiation between these different etiologies is difficult. The time after transplantation of occurrence of new neurological symptoms seems to be indicative. CNI‐induced neurological symptoms generally occur shortly after transplantation (days up to months).^19^, ^25^ This is confirmed in this study (all within 3 months after transplantation), while symptoms occurring more than a year after transplantation seem to be more likely metabolically induced, among other probably due to the entrapment of mma in the brain.^26^ To gain more insight in the effect of transplantation on mma levels in CSF and to aid clinical interpretation of new neurological symptoms after transplantation, we recommend the measurement of mma CSF levels before/during and after transplantation in all to be transplanted MMA patients.

However, in non‐MMA patients CNI‐induced PRES has also been described years after kidney transplantation^27^; and CNI‐induced neurotoxicity should therefore never be discarded as possible cause. CNI‐induced neurotoxicity frequently presents with seizures, both in transplanted MMA patients (5/7 with CNI‐induced neurotoxicity and 3/3 with PRES and in the majority of non‐MMA patients with CNI‐induced PRES).^15^, ^19^, ^28^ We recommend considering CNI‐induced neurotoxicity in all transplanted MMA patients presenting with new onset of seizures after transplantation. In CNI‐induced seizures more recently developed anticonvulsants should be used and no valproic acid.^15^

#### 4.1.2 Diagnosis and treatment

There are three steps to diagnose new neurological symptoms as CNI‐induced neurotoxicity (in which plasma CNI levels do not necessarily have to be above the therapeutic window,^25^, ^29^, ^30^ including PRES.^15^ First, other etiologies should be excluded, such as a metabolic stroke‐like episode, which is likely to be preceded by a systemic decompensation. Second, neuro‐imaging should be performed. In case T2 hyperintensity is found on MRI, DWI can help to distinguish between vasogenic edema, which is typically found in PRES, and cytotoxic edema (with restricted diffusion) that is typical for acute ischemic stroke. However, this is not always the case: restricted diffusion can also be encountered in PRES, and vasogenic edema can also occur in acute metabolic ischemic stroke. Brain location seemed typical only in one patient involving occipitoparietal region (Giussani). A recent study describes a central variant of PRES,^31^ which seems more in line with case 1. Third, there should be improvement of symptoms after reduction /discontinuation of the CNI. There are no clear recommendations on whether to reduce the dose or discontinue the CNI and how long discontinuation should last.^21^

#### 4.1.3 Outcome

With early recognition and treatment of CNI‐induced neurotoxicity, including PRES, the majority of patients will have no remaining neurological symptoms.^28^ However, life‐threatening complications may occur^32^ and acquired neurological damage may persist.^33^, ^34^ In the three MMA patients with PRES some remaining clinical abnormalities were found (case 1).^11^, ^24^

### 4.2 FGF‐21 and CNI‐induced neurotoxicity, including PRES, in transplanted MMA patients

The exact pathophysiology of CNI‐induced neurotoxicity, including PRES, is unclear but seems multifactorial.^35^, ^36^ Impairment of oxidative phosphorylation by CNIs and thereby mitochondrial dysfunction has been reported to be a possible cause.^37^, ^38^ Mitochondrial dysfunction plays an important role in the pathophysiology of MMA, and FGF‐21 is a biomarker for mitochondrial dysfunction. Clearly increased FGF‐21 levels have been described in MMA.^22^, ^39^, ^40^, ^41^, ^42^ We confirmed the severe initial decrease of FGF‐21 after transplantation as recently reported by Manoli et al^43^ in our two case reports. However, we also showed an increase in FGF‐21 plasma levels in case 1 at the time of neurological symptoms due to CNI‐induced PRES with a decrease in FGF‐21 with improvement of symptoms and brain MRI‐scan findings. This is suggestive for the pathogenic role of disturbed mitochondrial function in CNI‐induced PRES. In case 2, a severe increase of FGF‐21 was seen during hemorrhagic shock, which indicates that other factors, such as ischemia impair mitochondrial function as well. Importantly, further studies are required on the potential role of FGF‐21 in adding evidence to the underlying cause of CNI‐induced neurotoxicity in transplanted MMA patients with new neurological symptoms. We hypothesize that patients with pre‐existent mitochondrial dysfunction (as present in patients with MMA) are more at risk to develop CNI‐induced neurotoxicity than the general population because of the cumulative effect of CNI on the mitochondria. However, to date there is no clear evidence to support this and this should be prospectively investigated. If MMA patients indeed have a greater risk to develop CNI‐induced neurotoxicity than the general population we should consider to give a none CNI immunosuppressant, like Sirolimus, as first‐line immunosuppression in these patients, even though they are less potent as immunosuppressant.^44^, ^45^

### 4.3 Incidence of CNI‐induced neurotoxicity, including PRES, in non‐MMA transplanted patients

Previous studies in non‐MMA transplanted patients reported an incidence of CNI‐induced neurotoxicity in 20% of the transplanted adults^46^ and 8% of pediatric patients.^47^, ^48^ The incidence of CNI‐induced PRES has been reported to range from 0.34% to 1% of the non‐MMA patients after transplantation.^19^, ^25^, ^27^ The incidence of CNI‐induced neurotoxicity in transplanted MMA patients in this study seems comparable to the incidence with non‐MMA transplanted patients, while the incidence of PRES seems much higher than in non‐MMA transplanted patients.

### 4.4 Study limitations

Several limitations of this study require attention. The true incidence of CNI‐induced neurotoxicity is difficult to investigate by means of a systemic review since not all transplanted MMA patients have been published, neurological follow‐up was described only in a minority (20%) of the reported cases and it is uncertain whether the authors/clinicians were aware of the possibility of CNI‐induced neurotoxicity, including PRES, in MMA. Adding our patients to the total of transplanted patients could lead to bias. However, since these cases were the only two MMA patients transplanted within our center, we avoided this bias as much as possible. Furthermore, several authors published numerous articles with the likelihood of using the same patient case multiple times without explicitly mentioning so. We did therefore look into potential duplicates.

In order to investigate the true incidence of CNI‐induced neurotoxicity, including PRES, and neurological complications due to other causes it is essential to establish a thorough prospective database with follow‐up of all the MMA transplanted patients. Since pre‐existent MRI abnormalities are frequent in MMA patients a pre‐transplant brain MRI‐scan would be of great value to distinguish between pre‐existing and new problems in all (to be) transplanted MMA patients. In MMA patients typical MRI findings (with typical as well as atypical brain locations) suggest PRES with the added value of DWI and ADC (in first 10 days after new neurological symptoms). Furthermore, mma levels and FGF‐21 in plasma and cerebrospinal fluid should be included in future studies/databases.

## 5 CONCLUSION

CNI‐induced neurotoxicity should be considered in all transplanted MMA patients with CNI use and new neurological symptoms. When CNI‐induced neurotoxicity cannot be excluded brain MRI‐scans (including DWI) should be performed and CNI dose must be reduced or discontinued, since with timely recognition of CNI‐induced neurotoxicity and dose reduction or discontinuing CNI, symptoms can resolve.

## CONFLICT OF INTEREST

This research was performed independently of all financial sponsors other than Erasmus MC, University Medical Center

## AUTHOR CONTRIBUTIONS

F. M. participated in the study design, data analyses and data interpretation. She also drafted the manuscript and participated in the planning, conduct and reporting of the work described in the article. M.W. conceived the study, participated in its design and interpretation, and contributed to data interpretation and manuscript revision. She also participated in the planning, conduct and reporting of the work described in the article. J.L. participated in the design of the study. She also contributed to writing and revising the manuscript. S.D.‐M. participated in the design of the study. She also contributed to writing and revising the manuscript. J. van de W. participated in the design of the study. She also contributed to writing and revising the manuscript. E.J. participated in the design of the study. He also contributed to writing and revising the manuscript. W.O. participated in the design of the study. He also contributed to writing and revising the manuscript. E.B. participated in the design of the study. She also contributed to writing and revising the manuscript. A.W. van der E. participated in the design of the study. She also contributed to writing and revising the manuscript. She contributed to assess and report the radiological findings. M.W. conceived the study, participated in its design and interpretation, acted as principal investigator, and contributed to data interpretation and manuscript revision. She also participated in the planning, conduct and reporting of the work described in the article. M.W. serves as the guarantor for the article. All authors have read and approved the final version of the manuscript.

## ETHICS APPROVAL

The study was approved by the local ethics committee. All the procedures followed were in accordance with the ethical standards of the committee responsible for human experimentation (institutional and national) and with the Helsinki Declaration of 1975 as revised in 2000. Informed consent with regard to being included in the study was obtained from all patients or their legal guardians. This article contains no studies with animal subjects performed by any of the authors.

## Supporting information


**Figure S1** Brain magnetic resonance imaging (MRI) images of case 1 from our center. POD 83 = initial MRI or CT‐scan and POD 193 = follow‐up MRI. Post‐contrast images pons and cerebellum.

**Figure S2** Brain magnetic resonance imaging (MRI) images of case 2 from our center performed at POD 32. Vermis hypoplasia, enlarged fourth ventricle.

**Table S1** magnetic resonance imaging (MRI) abnormalities in those with CNI‐induced PRES. POD, postoperative day; Tx, transplantation.

**Table S2** A, Patients with reported neurologic follow‐up but without reported medication (n=5), which were probable CNI‐induced neurotoxicity. Abbreviations: n, number of patients; y, year; mo, month; POD, postoperative day; un, unavailable.
**Table S2B** Patients with reported neurologic follow‐up while on CNI (n = 2), which were probable CNI‐induced neurotoxicity. Abbreviations: n, number of patients; y, year; mo, month; POD, postoperative day; un, unavailable

**Table S3** A, Patients with neurotoxicity due to another cause without reported medication. Abbreviations: CNI, calcineurin inhibitor; LKT, liver and kidney transplant; LT, liver transplant; KT, kidney transplant; mo, months; n, number of patients; POD, postoperative day; y, reported time after transplant in years; un, unavailable.
**Table S3B** Patients with likely non‐CNI induced neurotoxicity with reported medication (CNI). Abbreviations: CNI, calcineurin inhibitor; LKT, liver and kidney transplant; LT, liver transplant; KT, kidney transplant; mo, months; n, number of patients; POD, postoperative day; y, reported time after transplant in years; un, unavailable
