# Supplementary material for: Neurotoxicity including posterior reversible encephalopathy syndrome after initiation of calcineurin inhibitors in transplanted methylmalonic acidemia patients: Two case reports and review of the literature
Source: JIMD Rep. 2020 Jan 22;51(1):89–104. doi: 10.1002/jmd2.12088 (PMC7012740; doi:10.1002/jmd2.12088)
Supplement: Supplementary file 3 — Table S1 magnetic resonance imaging (MRI) abnormalities in those with CNI‐induced PRES. POD, postoperative day; Tx, transplantation. [file JMD2-51-89-s003.docx]

| Case | Case 1  (see also figure 1) | Vernon | Giusanni |
| --- | --- | --- | --- |
| CNI start,  CNI stop,  CNI restart | POD1,  POD 86,  No | POD6,  POD28,  No | POD1,  POD10,  POD12 at lower dose |
| MRI/ CT-scan findings: | |  |  |
| Basal ganglia | Post Tx MRI: POD 83:  Symmetrical swelling, T2 hyperintensity, lentiform fork sign, high signal intensity on DWI with intermediate ADC, mild contrast enhancement  Follow-up Tx MRI: POD 193:  Decreased signal abnormalities, no more contrast enhancement, tissue loss | Post Tx MRI including MRA: POD 4:  Small T2-hyperintese lesions in the posterior aspect of the globi pallidi which were felt to be a sequelae of her prior infarct, unchanged on Follow-up Tx POD 28 |  |
| Mammillary bodies | Post Tx MRI: POD 83:  Symmetrical severe swelling and T2 hyperintensity, avid contrast enhancement, without diffusion restriction  Follow-up Tx MRI: POD 193:  Decreased signal abnormalities, no more contrast enhancement, tissue loss |  |  |
| Truncus cerebri | Post Tx MRI: POD 83:  T2 hyperintensity pons without diffusion restriction or contrast enhancement, probably due to postischemic changes  Follow-up Tx: POD 193:  conform | Follow-up Tx MRI: POD 28:  Mesencephalon and pons:  Patchy T2 hyperintense signal in the pons and left midbrain with matching increased apparent diffusion coefficient (ADC) values  Follow-up Tx MRI: POD 48:  Improvement in the previously visualized changes in the pons on T2 weighted images. |  |
| Cerebellum | Post Tx MRI: POD 83:  Symmetrical swelling, T2 hyperintensity, SCA territory without diffusion restriction.  Asymmetrical focal T2 hyperintensities with diffusion restriction PICA and AICA territory  Follow-up Tx MRI: POD 193:  Decreased signal abnormalities, atrophySCA area  Focal tissue losses PICA and AICA area without signal abnormalities of the remaining parenchyma |  |  |
| Cingulate gyrus |  | Follow-up MRI: POD 48:  There was minimally increased abnormal signal in the right cingulate gyrus with reduced diffusion on ADC maps |  |
| Diencephalon |  | Follow-up Tx MRI: POD 28:  Small T2 hyperintense lesions in the left thalamus without matching diffusion abnormalities  Follow-up Tx MRI: POD 48:  Improvement in the previously visualized changes in the thalamus on T2 weighted images |  |
| White matter |  | Follow-up Tx MRI: POD 28:  Small T2 hyperintense lesions left corona radiate without matching diffusion abnormalities | Post Tx CT: POD 10:  Subcortical hypodensities in the left temporal, right parietal, and bilateral occipital regions  Post Tx MRI: POD 10:  Multiple large areas of vasogenic edema in the occipitoparietal and frontotemporal regions on T2 and FLAIR images with no diffusion restriction |
| Capsula interna | Post Tx MRI and CT-scan: POD 83:  Anterior part calcifications  Follow-up Tx MRI: POD 193:  unchanged |  |  |
| Other: |  | Post Tx MRI: POD 4:  T2 hyperintense signal of the orbital segment of both optic nerves, and mild reduction in size of the right side of the optic chiasm, consistent with acute optic nerve damage superimposed on a chronic bilateral optic neuropathy |  |

Supplementary table 1. MRI abnormalities in those with CNI-induced PRES. Tx= transplantation, POD= postoperative day.
