# Supplementary material for: Neurotoxicity including posterior reversible encephalopathy syndrome after initiation of calcineurin inhibitors in transplanted methylmalonic acidemia patients: Two case reports and review of the literature
Source: JIMD Rep. 2020 Jan 22;51(1):89–104. doi: 10.1002/jmd2.12088 (PMC7012740; doi:10.1002/jmd2.12088)
Supplement: Supplementary file 4 — Table S2 A, Patients with reported neurologic follow‐up but without reported medication (n=5), which were probable CNI‐induced neurotoxicity. Abbreviations: n, number of patients; y, year; mo, month; POD, postoperative day; un, unavailable. Table S2B Patients with reported neurologic follow‐up while on CNI (n = 2), which were probable CNI‐induced neurotoxicity. Abbreviations: n, number of patients; y, year; mo, month; POD, postoperative day; un, unavailable [file JMD2-51-89-s004.docx]

| Case (n=) | Follow-up duration | | Age at transplantation | Time after transplantation and symptoms | MRI |
| --- | --- | --- | --- | --- | --- |
| Hoff (n=1) | *4y6mo* | *13y6mo* | | *<3month*  *Seizures and intracranial hemorrhage* | *un* |
| Kasahara (n=3) | *un* | *un* | | *un*  *New onset of seizures (3x)* | *un* |
| Yoshino (n=1) | *2y7mo* | *7y3m0* | | *POD 19*  *Episodes of quick torsional movements of head*  *(2y7mo*  *Developed an episode of tonic seizures)* | *un* |

Supplementary table 2a. Patients with reported neurologic follow-up but without reported medication (n=5), which were probable CNI-induced neurotoxicity. Abbreviations: n= number of patients; y=year; mo=month; un= unavailable; POD=postoperative day.

| Case (n=) | Follow-up duration | Age at transplantation (type of transplantation) | Time after transplantation and symptoms | MRI | Tacrolimus plasma levels | Outcome |
| --- | --- | --- | --- | --- | --- | --- |
| Kaplan (n=1) | *8y5mo* | *19mo (LT)* | *POD30*  *Severe, coarse, generalized tremors* | *POD 72*  *MRI: an acute lesion in the right globus pallidus, consistent with ischemic and/or edematous changes*  *Subsequent MRI 18 months later showed resolution of the basal ganglion lesion.* | *Within therapeutic range* | *Tacrolimus discontinued and cyclosporine started; no clear improvement of tremors (no information on time period given); subsequent reinstitution of tacrolimus and discontinuation of cyclosporine produced no immediate/apparent worsening (no information on time period given); treated with oral clonazepam therapy with gradual improvement. The tremors resolved after 1 year* |
| Burlina  (n=1) | *un* | *18y (KT)* | *4mo*  *Partial motor seizures with secondary generalization, without biochemical decompensation* | *DW-MRI: Hyperintense bilateral lesions tegmentum of the pons. MRI: confirmed the stroke-like lesions in the same areas* | *un* | *Antiepileptic therapy was started, no further outcome reported, no information on whether CNI was lowered or discontinued* |

Supplementary table 2b. Patients with reported neurologic follow-up while on CNI (n=2), which were probable CNI-induced neurotoxicity. Abbreviations: n= number of patients; y= year; mo=month; un=unavailable; POD=postoperative day
