# Supplementary material for: Neurotoxicity including posterior reversible encephalopathy syndrome after initiation of calcineurin inhibitors in transplanted methylmalonic acidemia patients: Two case reports and review of the literature
Source: JIMD Rep. 2020 Jan 22;51(1):89–104. doi: 10.1002/jmd2.12088 (PMC7012740; doi:10.1002/jmd2.12088)
Supplement: Supplementary file 5 — Table S3 A, Patients with neurotoxicity due to another cause without reported medication. Abbreviations: CNI, calcineurin inhibitor; LKT, liver and kidney transplant; LT, liver transplant; KT, kidney transplant; mo, months; n, number of patients; POD, postoperative day; y, reported time after transplant in years; un, unavailable. Table S3B Patients with likely non‐CNI induced neurotoxicity with reported medication (CNI). Abbreviations: CNI, calcineurin inhibitor; LKT, liver and kidney transplant; LT, liver transplant; KT, kidney transplant; mo, months; n, number of patients; POD, postoperative day; y, reported time after transplant in years; un, unavailable [file JMD2-51-89-s005.docx]

| Case (n=) | Current age of patient | Age at transplantation  (type of transplantation) | Time after transplantation and symptoms | CNI applied | MRI |
| --- | --- | --- | --- | --- | --- |
| Chakrapani (n=1) | *5y, 9mo* | *9mo*  *(LT)* | *NA (after surgery)*  *Seizures while having metabolic acidosis and hyperammonemia*  *5y*  *Had an unexpected episode with sudden onset of altered consciousness, loss of speech, and hypotonia.* | *un* | *un*  *CT unremarkable. Repeat neuroimaging 1 week after the acute episode revealed bilateral basal ganglia changes* |
| Khan (n=1) | *31y* | *Early in life*  *(2x LKT)* | *NA (at 20y of age)*  *Haemorrhaic stroke* | *un* | *un* |
| Shenoy (n=1) | *un* | *NA*  *(KT)* | *2 mo*  *Bacterial endocardititis and then progressive deterioration in graft function.*  *(13 mo*  *Episode of pancreatitis and neurological deterioration care was withdrawn)* | *un* | *un* |
| Nakajima (n=1) | *5y4mo* | *1y7mo*  *(LT)* | *1y7mo*  *Disability, altered consciousness, fever of unknown origin* | *un* | *MRI, MRS indicated Leigh’s encephalopathy* |
| Yoshino (n=1) | *5y2mo* | *2y*  *(LT)* | *2y*  *Weakness of right extremities and flexion of right upper extremity* | *un* | *un* |
| Sissaoui (n=1) | *un* | *un*  *(LKT)* | *NA*  *Axonal neuropathy and myoclonus* | *un* | *un* |

Supplementary table 3a. Patients with neurotoxicity due to another cause without reported medication. Abbreviations: n=number of patients; LT = liver transplant; KT= kidney transplant; LKT= liver and kidney transplant; CNI= calcineurin inhibitor; y = reported time after transplant in years; mo=months; POD = postoperative day; un = unavailable.

| Case (n=1) | Current age of patient | Age at transplantation  (organ) | Time after transplantation and symptoms | CNI applied | MRI |
| --- | --- | --- | --- | --- | --- |
| Clothier (n=1) | *16y* | *12y*  *(KT)* | *un*  *Generalized sensory motor peripheral neuropathy,* | *yes* | *MRI scans of his brain at ages 14 and 16 revealed long-standing bilateral changes in the globus pallid and no other significant abnormalities* |
| Khanna (n=1) | *28y* | *24y*  *(LT)* | *POD 183-191*  *Had a seizure episode and purulent meningitis* | *yes* | *un* |
| Brassier (n=1) | *8y* | *11y* | *18mo*  *Neurological regression with hepatoblastoma* | *yes* | *Brain MRI showed bilateral pallidal lesions, hypersignal of dentae nuclei, vermian atrophy and cerebral edema* |
| Kasahara (n=1) | *un* | *9mo*  *(LT)* | *5y*  *Severe neurological insult while recovering from chest infection without any systemic disturbance* | *yes* | *un* |
| Kasahara (n=2) | *un* | *22y and 13y respectively*  *(both LT)* | *un*  *Both progressive neurological disability* | *yes (2x)* | *un* |
| Nyhan (n=1) | *un* | *22y* | *un*  *Progressive neurologic abnormality; developed acute spasmodic contractions* | *yes* | *CT and MRI of the brain were normal; there were no lesions in the basal ganglia or white matter.* |

Supplementary table 3b. Patients with likely non-CNI induced neurotoxicity with reported medication (CNI). Abbreviations: n=number of patients; LT = liver transplant; KT= kidney transplant; LKT= liver and kidney transplant; CNI= calcineurin inhibitor; y= reported time after transplant in years; mo=months; POD, postoperative day; un = unavailable.
